# Synthesis and In Vitro Anticancer Evaluation of Flavone—1,2,3-Triazole Hybrids

**DOI:** 10.3390/molecules28020626

**Published:** 2023-01-07

**Authors:** Alexandra Németh-Rieder, Péter Keglevich, Attila Hunyadi, Ahmed Dhahir Latif, István Zupkó, László Hazai

**Affiliations:** 1 Department of Organic Chemistry and Technology, Faculty of Chemical Technology and Biotechnology, Budapest University of Technology and Economics, Műegyetem rkp. 3., H-1111 Budapest, Hungary; 2 Institute of Pharmacognosy, University of Szeged, Eötvös u. 6., H-6720 Szeged, Hungary; 3 Department of Pharmacodynamics and Biopharmacy, University of Szeged, Eötvös Str. 6, H-6720 Szeged, Hungary; 4 Department of Pharmacology and Toxicology, Faculty of Medicine, Wasit University, Wasit 52001, Iraq

**Keywords:** flavones, chrysin, kaempferol, hybrids, 1,2,3-triazole, anticancer activity

## Abstract

Hybrid compounds of flavones, namely chrysin and kaempferol, and substituted 1,2,3-triazole derivatives, were synthesized by click reaction of the intermediate *O*-propargyl derivatives. 4-Fluoro- and 4-nitrobenzyl-1,2,3-triazole-containing hybrid molecules were prepared. The mono- and bis-coupled hybrids were investigated on 60 cell lines of 9 common cancer types (NCI60) in vitro as antitumor agents. Some of them proved to have a significant antiproliferative effect.

## 1. Introduction

Cancer treatment is one of the most important medical challenges. Permanent research is in progress to produce more effective and less toxic derivatives. One of the exciting and promising directions of this research is the synthesis of antitumor hybrid molecules [1,2]. The concept of molecular hybridization is to incorporate two or more pharmacophores into one molecule with covalent bonds, increasing the chance of effectiveness and improving the drug kinetic properties of the resulting hybrid compared to the corresponding fixed-dose drug combination. It should be noted that rigid distance imposed by the structure of the compound between potentially active parts of the hybrid may prevent biological efficiency.

During our previous work, numerous new molecules exerting a significant antiproliferative effect have been developed in this field. Various hybrids of *Vinca* alkaloids [3] were synthesized, coupling with amino acid esters [4,5], steroids [6], flavones (e.g., **3**, chrysin) [7], phosphorus derivatives [8], amines [5], and compounds containing the known pharmacophore 1,2,3-triazole (**2**) [5]. Recently our work was extended to the synthesis of new aminochrysin derivatives coupled with different aromatics [9].

**Figure 1:**
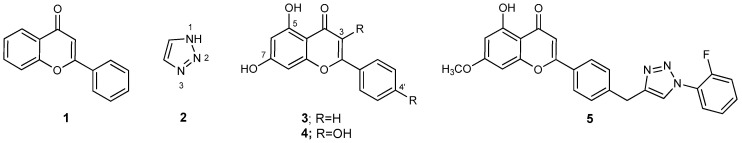
The structure of 2-phenylchromen-4-one (**1**), 1,2,3-triazole (**2**), chrysin (**3**), kaempferol (**4**), and an anticancer flavone—1,2,3-triazole hybrid (**5**).

Several flavonoids with antitumor activity are known in the literature [10,11]. Flavones containing a 2-phenylchromen-4-one (**1**) backbone, and 1,2,3-triazole derivatives keep attracting much research interest, and many 1,2,3-triazole-containing hybrids are known as effective anticancer agents [12,13,14,15]. During the last decade, numerous biologically active flavone—1,2,3-triazole hybrids have been synthesized [16,17,18], for example, **5** apigenin-7-methyl ether derivative, which showed promising activity against ovarian cancer (*IC*_50_ = 10, 15 and 20 µM for SKOV3, OVCAR-3 and Caov-3 cancer cell lines) (Figure 1) [19].

In this study, the above outlined results inspired us to develop synthetic possibilities for the preparation of flavone—1,2,3-triazole hybrids.

## 2. Results and Discussion

In the course of elaborating the synthetic design, chrysin (5,7-dihydroxyflavone) (**3**) and kaempferol (3,4′,5,7-tetrahydroxyflavone) (**4**) were chosen (Figure 1) to couple with 1,2,3-triazole derivatives. Some chrysin—1,2,3-triazole hybrids prepared by a different way, were previously reported as antibacterial agents [20].

### 2.1. Coupling Components

Chrysin (**3**) is among the best-known flavones. It is abundant in nature and present in many edible plants and honey [21]. It has an anticancer effect through inducing apoptosis and autophagy [21,22]. Chrysin (**3**) seems to be suitable for use alone and/or in combination with other chemotherapeutic agents [21]. Kaempferol (**4**) and its derivatives are also found in many plants. They can prevent coronary heart disease and inflammatory problems, and they also show antiproliferative effects and may induce apoptosis [23].

It is known that 1,2,3-Triazole derivatives have been widely used as a pharmacophore in hybrids. In addition to the advantageous physico-chemical properties of this moiety, it is also known to exert various biological effects [24,25]. 1,2,3-Triazole derivatives are characterized by stability, the ability to form hydrogen bonds (increasing their water solubility), and weak basicity (they are not protonated at physiological pH). Moreover, 1,2,3-triazole derivatives have fungicidal, antibacterial, antituberculosis, and anticancer effects [26,27]. The well-known click reaction is used for the preparation of 1,2,3-triazole derivatives, as one of the tools of modern organic synthetic methods based on structure-activity relationships, preferably the *N*^1^-(4-fluoro- and 4-nitrobenyzl)-1,2,3-triazole derivatives [24,28].

### 2.2. Chemistry

**Scheme 1:**
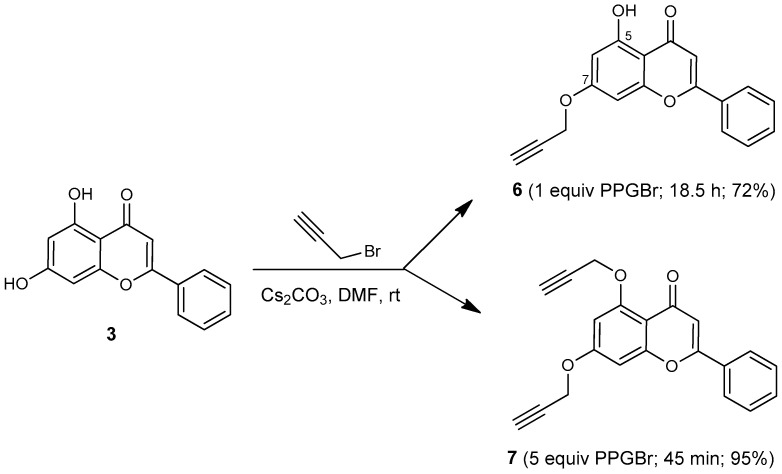
The reaction between chrysin (**3**) and propargyl bromide (PPGBr).

Chrysin (**3**) reacted with an equimolar quantity of propargyl bromide (PPGBr) in dimethylformamide in the presence of cesium carbonate at room temperature (Scheme 1), resulting in the 7-substituted product (**6**) (known as an intermediate of antibacterial derivative prepared by a method different from ours [20]). The reason for the regioselectivity is that the proton of the 5-hydroxyl group forms an intramolecular H-bond with the neighboring oxo group. Certainly, with an excess of propargyl bromide (5 equivalent), exclusively the 5,7-disubstituted derivative (**7**) proved to be the product, as expected. Others also synthesized this compound using gold(I) complexes without reporting any preparative and characterization details [29].

**Scheme 2:**
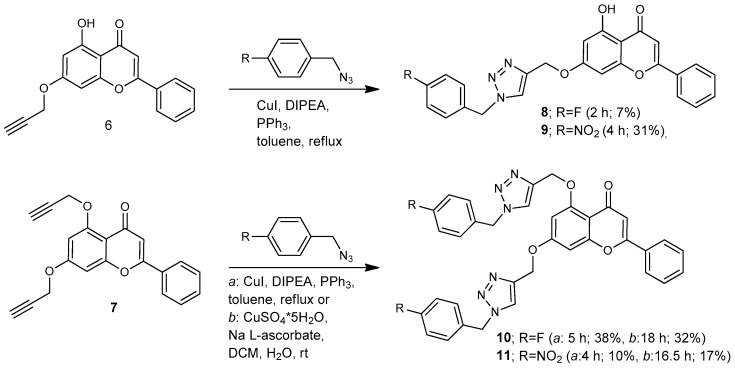
The synthesis of chrysin hybrids (**8**–**11**) containing one or two 1,2,3-triazole units.

The next reaction step was the click reaction (Scheme 2) using 4-fluoro- and 4-nitrobenzyl azide prepared in situ from the corresponding benzyl bromides with sodium azide in DMF at room temperature [30].

The reaction was carried out in the presence of copper(I) iodide, triphenylphosphine, and *N*,*N*-diisopropylethylamine, and resulted in known hybrids **8** and **9**, respectively. These two hybrids were prepared previously with another method, however, only their antibacterial effect has been investigated [20]. Bis(propargyl) derivative **7** was also treated with the same reaction conditions and gave the bis-hybrids **10** and **11**. Avoiding the difficult isolation from the triphenylphosphine oxide formed, the latter click reaction was successfully achieved also with further reagents, namely with copper sulfate pentahydrate and sodium L-ascorbate in a two-phase mixture.

**Scheme 3:**
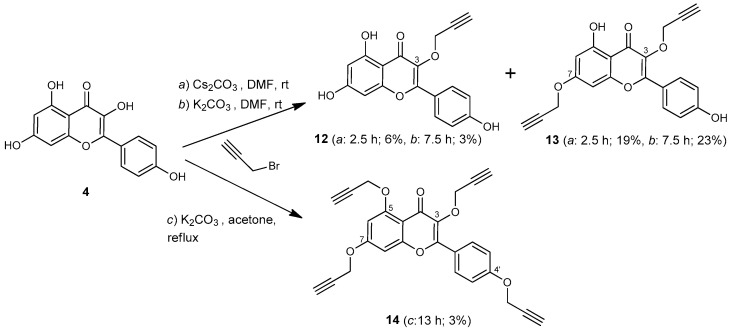
The reaction between kaempferol (**4**) and propargyl bromide.

The second flavone building block, selected for the synthesis of hybrids, was kaempferol (**4**). The alkylation with propargyl bromide was investigated with different bases and in different solvents (Scheme 3). Using cesium carbonate or potassium carbonate as a base in dimethylformamide compounds **12** and **13** were isolated. However, in acetone solution compound **14** was obtained.

**Scheme 4:**
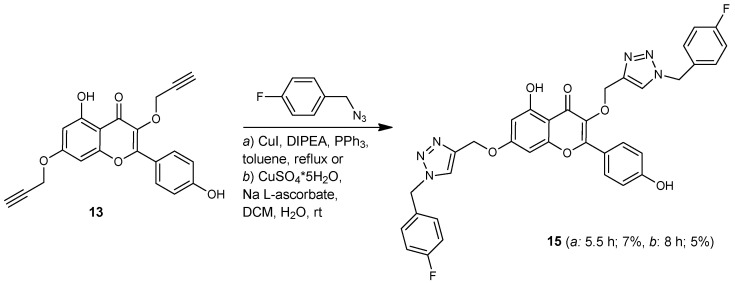
The synthesis of a kaempferol hybrid (**15**) containing two 1,2,3-triazole units.

Derivative **13** was the compound isolated in the relatively largest quantity and was chosen for the click reaction (Scheme 4). Investigating both the reaction conditions resulted in the isolation of the bis-hybrid **15**.

### 2.3. Biological Evaluation

The in vitro antiproliferative activities of chrysin (**3**) and the synthesized compounds (**8**–**11**, **15**) were examined against 60 human tumor cell lines according to the given protocols of NCI (USA) [31,32,33,34,35]. The results are summarized in Table 1. The percentages of growth show the amount of living cancer cells compared to a reference. The negative numbers indicate a significant decrease in the cell number. Since derivatives **8** and **10** had shown remarkable antiproliferative activity on several cancer cell lines during the one-dose test, they were subjected to a five-dose screening. The *GI*_50_ (50% growth inhibition) values are also given in Table 1.

It can be seen from Table 1 that no antiproliferative effect was shown by chrysin (3) and compounds **9** and **11**. Hybrids 8 and 10 cause cell death on several cell lines of different types of cancer and show inhibition effect also on some cases. Despite the relatively limited structural diversity of our compounds, the above results revealed some interesting structure-activity relationships. We found that (i) the bis-hybrid compounds also exert considerable antiproliferative effect and (ii) replacement of the fluorine atom by a nitro group reduces the bioactivity. The kaempferol-triazole hybrid (15) gave rather modest results.

The two promising compounds (**8** and **10**) were tested for their antiproliferative activity on further two human cervical cancer cell lines HeLa and SiHa (Table 2). Interestingly, the monohybrid derivative 8 was active only against HeLa cells, and SiHa cells were relatively resistant to it. The bis-hybrid derivative 10 was more potent and similarly active against both cell lines, with a sub-micromolar IC_50_ value against HeLa. Both derivatives exhibited higher activity than the reference agent cisplatin against HeLa cells.

The results obtained in this paper are encouraging for the future optimization of the derivatives. We want to emphasize that this study may be the starting point for more detailed synthetic and anticancer research.

## 3. Materials and Methods

### 3.1. General Materials and Methods

All chemicals were purchased from Sigma-Aldrich (Budapest, Hungary) and were used as received. Melting points were measured on a VEB Analytik Dresden PHMK-77/1328 apparatus (Dresden, Germany) and are uncorrected. IR spectra were recorded on Zeiss IR 75 and 80 instruments (Thornwood, NY, USA). NMR measurements were performed on a Bruker Avance III HDX 500 MHz NMR spectrometer equipped with a ^1^H{^13^C/^15^N} 5 mm TCI CryoProbe (Bruker Corporation, Billerica, MA, USA). ^1^H And ^13^C chemical shifts are given on the delta scale as parts per million (ppm) relative to tetramethyl silane. One-dimensional ^1^H, and ^13^C spectra and two-dimensional ^1^H–^1^H COSY, ^1^H–^1^H NOESY, ^1^H–^13^C HSQC, and ^1^H–^13^C HMBC spectra were acquired using pulse sequences included in the standard spectrometer software package (Bruker TopSpin 3.5, Bruker Corporation). ESI-HRMS and MS-MS analyses were performed on a Thermo Velos Pro Orbitrap Elite (Thermo Fisher Scientific, Bremen, Germany) system. The ionization method was ESI, operated in positive ion mode. The protonated molecular ion peaks were fragmented by CID (collision-induced dissociation) at a normalized collision energy of 35–65%. For the CID experiment, helium was used as the collision gas. The samples were dissolved in methanol. EI-HRMS analyses were performed on a Thermo Q Exactive GC Orbitrap (Thermo Fisher Scientific, Bremen, Germany) system. The ionization method was EI and operated in positive ion mode. Electron energy was 70 eV and the source temperature was set at 250 °C. Data acquisition and analysis were accomplished with Xcalibur software version 4.0 (Thermo Fisher Scientific). TLC was carried out using DC-Alufolien Kieselgel 60 F_254_ (Merck, Budapest, Hungary) plates. Preparative TLC analyses were performed on silica gel 60 PF_254+366_ (Merck) glass plates.

### 3.2. Chemistry

#### 3.2.1. 7-(*O*-Propargyl)chrysin (**6**)

Chrysin (**3**) (330 mg, 1.3 mmol) and cesium carbonate (426 g, 1.3 mmol) were dissolved in DMF (15 mL), the solution was stirred at 10 min, then propargyl bromide (0.142 mL, 1.3 mmol) was added in 80% toluene solution. After stirring at room temperature for 18.5 hrs, the reaction mixture was evaporated to dryness, and the residue was dissolved in dichloromethane (60 mL). Next, water (60 mL) was added, and the pH was adjusted to 1 with 2*M* hydrochloric acid solution. The water phase was extracted with dichloromethane (2 × 30 mL), then the combined organic phase was washed with water (60 mL) and saturated sodium chloride solution (60 mL). The organic phase after drying with magnesium sulfate was evaporated to dryness, and the crude product was purified with preparative TLC (dichloromethane-methanol = 40:1) to give 274 mg (72%) of compound **6** as a yellow solid. M.p.:180–182 °C. TLC (dichloromethane-methanol = 30:1); *R_f_* = 0.83. IR (KBr) 3284, 1663, 1624, 1540, 1331, 1155, 767 cm^−1^. ^1^H NMR (499.9 MHz; DMSO-*d*_6_) *δ* (ppm) 3.69 (t; *J* = 2.4 Hz; 1H; C(7)-OCH_2_C≡CH); 4.97 (d; *J* = 2.4 Hz; 2H; C(7)-OCH_2_); 6.48 (d; *J* = 2.3 Hz; 1H; H-6); 6.88 (d; *J* = 2.3 Hz; 1H; H-8); 7.07 (s; 1H; H-3); 7.58–7.62 (m; 2H; H-3′, H-5′); 7.62–7.66 (m; 1H; H-4′); 8.09–8.13 (m; 2H; H-2′, H-6′); 12.84 (s; 1H; C(5)-OH). ^13^C NMR (125.7 MHz; DMSO-*d*_6_) *δ* (ppm) 56.2 (C(7)-OCH_2_); 78.3 (C(7)-OCH_2_C≡CH); 79.0 (C(7)-OCH_2_C≡CH); 93.7 (C-8); 98.6 (C-6); 105.2 (C-10); 105.4 (C-3); 126.4 (C-2′, C-6′); 129.1 (C-3′, C-5′); 130.5 (C-1′); 132.1 (C-4′); 157.1 (C-9); 161.1 (C-5); 163.1 (C-7); 163.5 (C-2). 182.1 (C-4). ESI-HRMS:M + H = 293.08086 (delta = 0.08 ppm; C_18_H_13_O_4_). HR-ESI-MS-MS (CID = 55%; rel. int. %): 269(5); 265(100); 251(56); 247(4); 239(6); 223(10).

#### 3.2.2. 5,7-Bis(*O*-propargyl)chrysin (**7**)

Chrysin (**3**) (500 mg, 1.97 mmol) and cesium carbonate (3.2 g, 9.84 mmol) were dissolved in dimethylformamide (20 mL), the solution was stirred at 10 min, then propargyl bromide (1.1 mL, 9.84 mmol) was added in 80% toluene solution. After stirring at room temperature for 45 min, the reaction mixture was evaporated to dryness, and the residue was dissolved in dichloromethane (40 mL). Next, water (40 mL) was added, and the pH was adjusted to 1 with 2*M* hydrochloric acid solution. The water phase was extracted with dichloromethane (3 × 20 mL), then the combined organic phase was washed with water (2 × 20 mL) and saturated sodium chloride solution (20 mL). The organic phase after drying with magnesium sulfate was evaporated to dryness and 620 mg (95%) pure product (**7**) was obtained. M.p.: 204–206 °C. TLC (dichloromethane-methanol = 30:1); *R_f_* = 0.33. IR (KBr) 3214, 1635, 1597, 1450, 1343, 1164, 833 cm^−1^. ^1^H NMR (499.9 MHz; DMSO-*d*_6_) *δ* (ppm) 3.63 (t; *J* = 2.4 Hz; 1H; C(5)-OCH_2_C≡CH); 3.70 (t; *J* = 2.4 Hz; 1H; C(7)-OCH_2_C≡CH); 4.93 (d; *J* = 2.4 Hz; 2H; C(5)-OCH_2_); 4.98 (d; *J* = 2.4 Hz; 2H; C(7)-OCH_2_); 6.67 (d; *J* = 2.3 Hz; 1H; H-6); 6.82 (s; 1H; H-3); 7.00 (d; *J* = 2.3 Hz; 1H; H-8); 7.54–7.62 (m; 3H; H-3′, H-4′, H-5′); 8.01–8.09 (m; 2H; H-2′, H-6′). ^13^C NMR (125.7 MHz; DMSO-*d*_6_) *δ* (ppm) 56.2 (C(7)-OCH_2_); 56.4 (C(5)-OCH_2_); 78.3 (C(7)-OCH_2_C≡CH); 78.6 (C(5)-OCH_2_C≡CH); 78.8 (C(5)-OCH_2_C≡CH); 79.0 (C(7)-OCH_2_C≡CH); 95.0 (C-8); 98.8 (C-6); 108.2 (C-3); 109.1 (C-10); 125.9 (C-2′, C-6′); 129.0 (C-3′, C-5′); 130.7 (C-1′); 131.4 (C-4′); 157.8 (C-5); 158.8 (C-9); 159.7 (C-2); 161.2 (C-7); 175.4 (C-4). ESI-HRMS: M + H = 331.09618 (delta = −0.9 ppm; C_21_H_15_O_4_). HR-ESI-MS-MS (CID = 35%; rel. int. %): 313(6); 303(89); 292(47); 289(16); 275(13); 265(13); 251(100); 185(36); 157(10).

#### 3.2.3. Click Reaction of 7-(*O*-Propargyl)chrysin (**6**) with 4-Fluorobenzyl Azide; Preparation of **8**

To 7-*O*-propargyl chrysin (**6**) (48 mg, 0.164 mmol) was added 4-fluorobenzyl azide (25 mg, 0.164 mmol) in toluene solution (4 mL) prepared in situ [30], triphenylphosphine (9 mg, 0.0328 mmol), copper(I) iodide (4 mg, 0.0164 mmol) and 0.09 mL (0.492 mmol) diisopropylethylamine. After reflux for 2 h, the reaction mixture was diluted with toluene (25 mL), then the mixture was washed with water (30 mL). After washing the water phase with toluene (10 mL), the combined organic phase after drying with magnesium sulfate was evaporated to dryness. The preparative TLC (dichloromethane-methanol = 40:1) of the crude product resulted in 5 mg (7%) pure product (**8**). M.p.:163–165 °C. M.p. *lit*.: 190-191^o^C [20]. TLC (dichloromethane-methanol = 40:1); *R_f_*=0.34. IR (KBr) 3424, 1660, 1614, 1558, 1161, 766, 541 cm^−1^. ^1^H NMR (499.9 MHz; DMSO-*d*_6_) *δ* (ppm) 5.29 (s; 2H; H_2_-1′); 5.62 (s; 2H; H_2_-7′); 6.50 (d; *J* = 2.2 Hz; 1H; H-6); 6.96 (d; *J* = 2.2 Hz; 1H; H-8); 7.06 (s; 1H; H-3); 7.19–7.24 (m; 2H; H-10′, H-12′); 7.39–7.44 (m; 2H; H-9′, H-13′); 7.58–7.66 (m; 3H; C(2)-Ph: 2x H*_meta_*, H*_para_*); 8.09–8.12 (m; 2H; C(2)-Ph: 2x H*_ortho_*); 8.35 (s; 1H; H-6′); 12.8 (br; 1H; C(5)-OH). ^13^C NMR (125.7 MHz; DMSO-*d*_6_) *δ* (ppm) 52.0 (C-7′); 61.7 (C-1′); 93.5 (C-8); 98.6 (C-6); 105.0 (C-10); 105.3 (C-3); 115.5 (d; ^2^*J*_CF_ = 21.6 Hz; C-10′, C-12′); 124.9 (C-6′); 126.4 (C(2)-Ph: C*_ortho_*); 129.1 (C(2)-Ph: C*_meta_*); 130.3 (d; ^3^*J*_CF_ = 8.5 Hz; C-9′, C-13′); 130.5 (C(2)-Ph: C_ipso_); 132.0–132.1 (m; C-8′, C(2)-Ph: C*_para_*); 142.0 (C-2′); 157.2 (C-9); 161.1 (C-5); 161.8 (d; ^1^*J*_CF_ = 244.2 Hz; C-11′); 163.5 (C-2); 163.9 (C-7); 182.0 (C-4). ESI-HRMS: M+H = 444.13547 (delta = 0.13 ppm; C_25_H_19_O_4_N_3_F). HR-ESI-MS-MS (CID = 45%; rel. int. %): 416(100); 363(32); 307(24); 293(12); 291(60); 267(26); 255(47).

#### 3.2.4. Click Reaction of 7-(*O*-Propargyl)chrysin (**6**) with 4-Nitrobenzyl Azide; Preparation of **9**

To 7-*O*-propargyl chrysin (**6**) (48 mg, 0.164 mmol) was added 4-nitrobenzyl azide (29 mg, 0.164 mmol) in toluene solution (4 mL) prepared in situ [30], triphenylphosphine (9 mg, 0.0328 mmol), copper(I) iodide (4 mg, 0.0164 mmol) and 0.09 mL (0.492 mmol) diisopropylethylamine. After reflux for 4 h, the reaction mixture was diluted with toluene (25 mL), then the mixture was washed with water (30 mL). After washing the water phase with toluene (10 mL), the combined organic phase after drying with magnesium sulfate was evaporated to dryness. The residue was dissolved in dichloromethane and after filtration, the filtrate was evaporated to dryness, then 24 mg (31%) product (**9**) was obtained. M.p.: 219–221 °C. M.p*. lit*.: 187–188 °C [20]. TLC (dichloromethane-methanol = 40:1); *R_f_* = 0.45. IR (KBr) 809; 1155; 1349; 1524; 1617; 1656; 3083 cm^−1^. ^1^H NMR (499.9 MHz; DMSO-*d*_6_) *δ* (ppm) 5.32 (s; 2H; H_2_-1′); 5.83 (s; 2H; H_2_-7′); 6.51 (d; *J* = 2.1 Hz; 1H; H-6); 6.97 (d; *J* = 2.1 Hz; 1H; H-8); 7.07 (s; 1H; H-3); 7.53–7.58 (m; 2H; H-9′, H-13′); 7.58–7.67 (m; 3H; C(2)-Ph: 2x H*_meta_*, H*_para_*); 8.07–8.13 (m; 2H; C(2)-Ph: 2x H*_ortho_*); 8.22–8.27 (m; 2H; H-10′, H-12′); 8.43 (s; 1H; H-6′); 12.83 (s; 1H; C(5)-OH). ^13^C NMR (125.7 MHz; DMSO-*d*_6_) *δ* (ppm) 51.9 (C-7′); 61.7 (C-1′); 93.6 (C-8); 98.7 (C-6); 105.1 (C-10); 105.4 (C-3); 123.9 (C-10′, C-12′); 125.4 (C-6′); 126.4 (C(2)-Ph: C*_ortho_*); 129.0 (C-9′, C-13′); 129.1 (C(2)-Ph: C*_meta_*); 130.5 (C(2)-Ph: C_ipso_); 132.1 (C(2)-Ph: C*_para_*); 142.2 (C-2′); 143.2 (C-8′); 147.2 (C-11′); 157.2 (C-9); 161.1 (C-5); 163.5 (C-2); 163.9 (C-7); 182.0 (C-4). ESI-HRMS: M+H=471.12976 (delta = −0.3 ppm; C_25_H_19_O_6_N_4_). HR-ESI-MS-MS (CID=35%; rel. int. %): 443(25); 425(12); 307(18); 291(26); 255(100); 189(2).

#### 3.2.5. Click Reaction of 5,7-Bis(*O*-propargyl)chrysin (**7**) with 4-Fluorobenzyl Azide; Preparation of **10**

**Figure 2:**
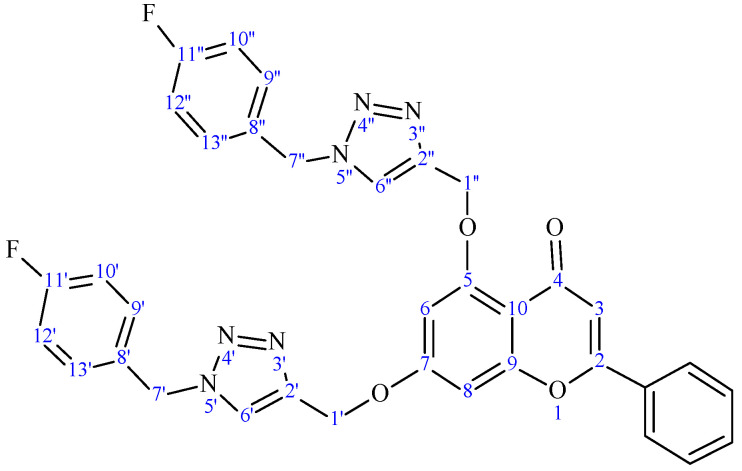
The skeleton numbering of compound **10** used for NMR assignment.

(a) To 5,7-bis(*O*-propargyl) chrysin (**7**) (44 mg, 0.133 mmol) was added 4-fluorobenzyl azide (40 mg, 0.265 mmol) in toluene solution (6 mL) prepared in situ [30], triphenylphosphine (14 mg, 0.0532 mmol), copper(I) iodide (5 mg, 0.0265 mmol) and 0.14 mL (0.798 mmol) diisopropylethylamine. After reflux for 5 hrs, the reaction mixture was diluted with toluene (25 mL), and the mixture was washed with water (30 mL), then the water phase was washed with toluene (10 mL). The combined organic phase was dried with magnesium sulfate and the precipitated product (**10**) (32 mg, 38%) could be separated with filtration.

(b) To 5,7-bis(*O*-propargyl) chrysin (**7**) (44 mg, 0.133 mmol) was added 4-fluorobenzyl azide (40 mg, 0.266 mmol) in dichloromethane solution (4.5 mL) prepared in situ [30], copper(II) sulfate pentahydrate (56 mg, 0.222 mmol), sodium L-ascorbate (88 mg, 0.443 mmol) and water (4.5 mL). After 18 hrs of intensive stirring at room temperature, the reaction mixture was diluted with water (18 mL) and extracted with dichloromethane (2 × 20 mL). The combined organic phase was washed with saturated sodium chloride solution (50 mL), and after drying with magnesium sulfate the solution was evaporated. The residue was separated with preparative TLC (dichloromethane-methanol = 15:1) and 27 mg (32%) product (**10**) was obtained (Figure 2). Mp.: 229–231 °C. TLC (dichloromethane-methanol = 20:1); *R_f_* = 0.30. IR (KBr) 1642, 1605, 1511, 1352, 1225, 1159 cm^−1^. ^1^H NMR (499.9 MHz; DMSO-*d*_6_) *δ* (ppm) 5.23 (s; 2H; H_2_-1″); 5.31 (s; 2H; H_2_-1′); 5.63 (s; 2H; H_2_-7′); 5.64 (s; 2H; H_2_-7″); 6.76 (s; 1H; H-3); 6.80 (d; *J* = 2.2 Hz; 1H; H-6); 7.07 (d; *J* = 2.2 Hz; 1H; H-8); 7.18–7.24 (m; 4H; H-10′, H-12′, H-10″, H-12″); 7.38–7.42 (m; 2H; H-9″, H-13″); 7.40–7.45 (m; 2H; H-9′, H-13′); 7.54–7.61 (m; 3H; C(2)-Ph: 2x H*_meta_*, H*_para_*); 8.02–8.06 (m; 2H; C(2)-Ph: 2x H*_ortho_*); 8.31 (s; 1H; H-6′’); 8.37 (s; 1H; H-6′). ^13^C NMR (125.7 MHz; DMSO-*d*_6_) *δ* (ppm) 51.9 (C-7″); 52.0 (C-7′); 61.6 (C-1′); 62.6 (C-1′’); 94.8 (C-8); 98.4 (C-6); 108.2 (C-3); 108.8 (C-10); 115.4–115.6 (m; C-10′, C-12′, C-10″, C-12′’); 124.5 (C-6″); 124.9 (C-6′); 125.8 (C(2)-Ph: C*_ortho_*); 129.0 (C(2)-Ph: C*_meta_*); 130.2 (d; ^3^*J*_CF_ = 8.5 Hz; C-9″, C-13″); 130.3 (d; ^3^*J*_CF_ = 8.5 Hz; C-9′, C-13′); 130.7 (C(2)-Ph: C_ipso_); 131.4 (C(2)-Ph: C*_para_*); 132.1–132.2 (m; C-8′, C-8″); 142.1 (C-2′); 142.9 (C-2′’); 158.7 (C-5); 159.0 (C-9); 159.6 (C-2); 161.78 (d; ^1^*J*_CF_ = 244 Hz), 161.81 (d; ^1^*J*_CF_ = 244 Hz): C-11′, C-11″; 162.2 (C-7); 175.4 (C-4). ESI-HRMS: M + H = 633.20636 (delta = 1.14 ppm; C_35_H_27_O_4_N_6_F_2_). HR-ESI-MS-MS (CID = 35%; rel. int. %): 605(43); 588(5); 577(6); 552(8); 498(8); 496(8); 456(5); 452(26); 444(48); 424(5); 399(6).

#### 3.2.6. Click Reaction of 5,7-Bis(*O*-propargyl)chrysin (**7**) with 4-Nitrobenzyl Azide; Preparation of **11**

(a) To 5,7-bis(propargyl) chrysin (**7**) (44 mg, 0.133 mmol) was added 4-nitrobenzyl azide (47 mg, 0.266 mmol) in toluene solution (6 mL) prepared in situ [30], triphenylphosphine (14 mg, 0.0532 mmol), copper(I) iodide (5 mg, 0.0265 mmol) and 0.14 mL (0.798 mmol) diisopropylethylamine. After reflux for 4 hrs, the reaction mixture was diluted with toluene (25 mL), and the mixture was washed with water (30 mL), then the water phase was washed with toluene (10 mL). The combined organic phase was dried with magnesium sulfate, and the precipitated crude product could be separated with filtration. After preparative TLC (dichloromethane-methanol = 15:1) of the crude product, 9 mg (10%) pure product (**11**) was obtained.

(b) To 5,7-bis(*O*-propargyl) chrysin (**7**) (176 mg, 0.532 mmol) was added 4-nitrobenzyl azide (190 mg, 1.064 mmol) in dichloromethane solution (18 mL) prepared in situ [30], copper(II) sulfate pentahydrate (224 mg, 0.888 mmol), sodium L-ascorbate (352 mg, 1.772 mmol) and water (18 mL). After 16.5 h of intensive stirring at room temperature, the reaction mixture was diluted with water (72 mL) and extracted with dichloromethane (3 × 80 mL). The combined organic phase was washed with saturated sodium chloride solution (200 mL), and after drying with magnesium sulfate the solution was evaporated. The residue was separated with preparative TLC (dichloromethane-methanol = 15:1) and 30 mg (17%) product (**11**) was obtained. M.p. = 182–184 °C. TLC (dichloromethane-methanol = 20:1); *R_f_* = 0.40. IR (KBr) 805; 1109; 1167; 1348; 1521; 1608; 1644; 3080 cm^−1^. ^1^H NMR (499.9 MHz; DMSO-*d*_6_) *δ* (ppm) 5.27 (s; 2H; H_2_-1′’); 5.34 (s; 2H; H_2_-1′); 5.83 (s; 2H; H_2_-7′); 5.84 (s; 2H; H_2_-7′’); 6.76 (s; 1H; H-3); 6.82 (d; *J* = 2.3 Hz; 1H; H-6); 7.09 (d; *J* = 2.3 Hz; 1H; H-8); 7.53–7.61 (m; 7H; H-9′, H-13′, H-9′’, H-13′’, C(2)-Ph: 2x H*_meta_*, H*_para_*); 8.02–8.06 (m; 2H; C(2)-Ph: 2x H*_ortho_*); 8.22–8.26 (m; 4H; H-10′, H-12′, H-10′’, H-12′’); 8.39 (s; 1H; H-6′’); 8.45 (s; 1H; H-6′). ^13^C NMR (125.7 MHz; DMSO-*d*_6_) *δ* (ppm) 51.8 (C-7′’); 51.9 (C-7′); 61.6 (C-1′); 62.6 (C-1′’); 94.9 (C-8); 98.4 (C-6); 108.2 (C-3); 108.8 (C-10); 123.8 (C-10′, C-12′, C-10′’, C-12′’); 125.0 (C-6′’); 125.4 (C-6′); 125.8 (C(2)-Ph: C*_ortho_*); 129.0 (C-9′, C-13′, C-9′’, C-13′’, C(2)-Ph: C*_meta_*); 130.7 (C(2)-Ph: C_ipso_); 131.4 (C(2)-Ph: C*_para_*); 142.2 (C-2′); 143.1 (C-2′’); 143.2 (C-8′); 143.3 (C-8′’); 147.2 (C-11′, C-11″); 158.7 (C-5); 159.0 (C-9); 159.6 (C-2); 162.1 (C-7); 175.4 (C-4). ESI-HRMS: M+H = 687.19286 (delta = −2.6 ppm; C_35_H_27_O_8_N_8_). HR-ESI-MS-MS (CID = 35%; rel. int. %): 659(45); 631(10); 507(84); 471(100); 443(12); 343(3); 291(7).

#### 3.2.7. *O*-Alkylation of Kaempferol (**4**) with Propargyl Bromide

(a) Kaempferol (**4**) (113 mg, 0.393 mmol) and cesium carbonate (129 mg, 0.393 mmol) were dissolved in dimethylformamide (5 mL) and after 10 min stirring propargyl bromide (0.043 mL, 0.393 mmol) was added in 80% toluene solution. The reaction mixture was stirred at room temperature for 2.5 hrs and was evaporated to dryness. The residue was dissolved in dichloromethane (20 mL), then water (20 mL) was added and the pH was adjusted to 1 with 2*N* hydrochloric solution. The water phase was washed with dichloromethane (2 × 10 mL), the combined organic phase was treated with water (20 mL), and then with saturated sodium chloride solution (20 mL). After drying with magnesium sulfate the solution was evaporated to dryness and using preparative TLC (dichloromethane-methanol = 20:1) two products were obtained: 8 mg (6%) of monopropargylated derivative (**12**), and 25 mg (19%) of 3,7-bis(*O*-propargyl) kaempferol (**13**).

(b) Kaempferol (**4**) (1130 mg, 3.93 mmol) and potassium carbonate (543 mg, 3.93 mg) were dissolved in dimethylformamide (15 mL). After 10 min stirring at room temperature propargyl bromide (0.43 mL, 3.93 mmol, in 80% toluene solution) dissolved in dimethylformamide (5 mL) was dropped into the reaction mixture. After 2.5 h further potassium carbonate (272 mg, 1.97 mmol) and propargyl bromide (0.22 mL, 1.97 mmol, in 80% toluene solution) dissolved in dimethylformamide (2 mL) were added. The reaction mixture was stirred for a further 5 hrs at room temperature, evaporated to dryness, and the residue was dissolved in chloroform (200 mL). Next, water (200 mL) was added, and the pH was adjusted to 1 with 2*N* hydrochloric acid. The water phase was extracted with chloroform (2x100 mL), and the combined organic phase was washed with water (200 mL) and with saturated sodium chloride solution (200 mL) and evaporated to dryness. Preparative TLC (dichloromethane-methanol = 20:1) separation of the residue 40 mg (3%) of **12** and 330 mg (23%) of **13** were obtained.

3-(*O*-Propargyl)kaempferol (**12**): M.p. = 180–182 °C. TLC (dichloromethane-methanol = 20:1); *R_f_* = 0.19. IR (KBr) 821; 1180; 1235; 1557; 1660; 3293 cm^−1^. ^1^H NMR (499.9 MHz; DMSO-*d*_6_) *δ* (ppm) 3.50 (t; *J* = 2.4 Hz; 1H; C(3)-OCH_2_C≡CH); 4.89 (d; *J* = 2.4 Hz; 2H; C(3)-OCH_2_); 6.22 (d; *J* = 2.1 Hz; 1H; H-6); 6.46 (d; *J* = 2.1 Hz; 1H; H-8); 6.90–6.95 (m; 2H; H-3′, H-5′); 7.98–8.01 (m; 2H; H-2′, H-6′); 10.28 (br s; 1H; C(4′)-OH); 10.90 (br; 1H; C(7)-OH); 12.55 (s; 1H; C(5)-OH). ^13^C NMR (125.7 MHz; DMSO-*d*_6_) *δ* (ppm) 58.8 (C(3)-OCH_2_); 78.6 (C(3)-OCH_2_C≡CH); 79.2 (C(3)-OCH_2_C≡CH); 93.7 (C-8); 98.6 (C-6); 103.8 (C-10); 115.4 (C-3′, C-5′); 120.4 (C-1′); 130.4 (C-2′, C-6′); 134.8 (C-3); 156.25 (C-9); 156.34 (C-2); 160.1 (C-4′); 161.1 (C-5); 164.2 (C-7); 177.6 (C-4). EI-HRMS: M = 324.06174 (delta = −3.4 ppm; C_18_H_12_O_6_).

3,7-Bis(*O*-propargyl)kaempferol (**13**): M.p. = 185–187 °C. TLC (dichloromethane-methanol = 20:1); *R_f_* = 0.49. IR (KBr) 1180; 1289; 1332; 1493; 1602; 1662; 3259 cm^−1^. ^1^H NMR (499.9 MHz; DMSO-*d*_6_) *δ* (ppm) 3.51 (t; *J* = 2.4 Hz; 1H; C(3)-OCH_2_C≡CH); 3.68 (t; *J* = 2.4 Hz; 1H; C(7)-OCH_2_C≡CH); 4.91 (d; *J* = 2.4 Hz; 2H; C(3)-OCH_2_); 4.95 (d; *J* = 2.4 Hz; 2H; C(7)-OCH_2_); 6.46 (d; *J* = 2.3 Hz; 1H; H-6); 6.82 (d; *J* = 2.3 Hz; 1H; H-8); 6.93–6.97 (m; 2H; H-3′, H-5′); 8.01–8.05 (m; 2H; H-2′, H-6′); 10.3–10.4 (br; 1H; C(4′)-OH); 12.55 (s; 1H; C(5)-OH). ^13^C NMR (125.7 MHz; DMSO-*d*_6_) *δ* (ppm) 56.2 (C(7)-OCH_2_); 58.9 (C(3)-OCH_2_); 78.3 (C(7)-OCH_2_C≡CH); 78.5 (C(3)-OCH_2_C≡CH); 79.0 (C(7)-OCH_2_C≡CH); 79.3 (C(3)-OCH_2_C≡CH); 93.3 (C-8); 98.4 (C-6); 105.2 (C-10); 115.4 (C-3′, C-5′); 120.3 (C-1′); 130.5 (C-2′, C-6′); 135.1 (C-3); 155.9 (C-9); 156.8 (C-2); 160.3 (C-4′); 160.8 (C-5); 162.9 (C-7); 177.8 (C-4). EI-HRMS: M = 362.07804 (delta = −1.2 ppm; C_21_H_14_O_6_).

(c) Kaempferol (**4**) (200 mg, 0.699 mmol) and potassium carbonate (106 mg, 0.769 mg) were dissolved in acetone (7 mL). After 10 min stirring at room temperature propargyl bromide (0.076 mL, 0.699 mmol, in 80% toluene solution) dissolved in acetone (3 mL) was dropped into the reaction mixture. After 7 hrs reflux further potassium carbonate (53 mg, 0.35 mmol) and propargyl bromide (0.038 mL, 0.35 mmol, in 80% toluene solution) dissolved in acetone (1.5 mL) were added. The reaction mixture was refluxed for a further 6 hrs, evaporated to dryness, and the residue was dissolved in dichloromethane (40 mL). Next, water (40 mL) was added and the pH was adjusted to 1 with 2*N* hydrochloric acid. The water phase was extracted with dichloromethane (2 × 20 mL), the combined organic phase was washed with water (40 mL) and with saturated sodium chloride solution (40 mL) and evaporated to dryness. After preparative TLC (dichloromethane-methanol = 20:1) separation of the residue, 10 mg (3%) of product **14** was obtained. M.p. = 158–160 °C. TLC (dichloromethane-methanol = 20:1); *R_f_* = 0.82. IR (KBr) 1174; 1185; 1509; 1605; 1627; 3287 cm^−1^. ^1^H NMR (499.9 MHz; DMSO-*d*_6_) *δ* (ppm) 3.47 (t; *J* = 2.4 Hz; 1H; C(3)-OCH_2_C≡CH); 3.64 (t; *J* = 2.4 Hz; 1H; C(4′)-OCH_2_C≡CH); 3.65 (t; *J* = 2.4 Hz; 1H; C(5)-OCH_2_C≡CH); 3.69 (t; *J* = 2.4 Hz; 1H; C(7)-OCH_2_C≡CH); 4.91 (d; *J* = 2.4 Hz; 2H; C(3)-OCH_2_); 4.92 (d; *J* = 2.4 Hz; 2H; C(4′)-OCH_2_); 4.93 (d; *J* = 2.4 Hz; 2H; C(5)-OCH_2_); 4.97 (d; *J* = 2.4 Hz; 2H; C(7)-OCH_2_); 6.64 (d; *J* = 2.3 Hz; 1H; H-6); 6.96 (d; *J* = 2.3 Hz; 1H; H-8); 7.15–7.19 (m; 2H; H-3′, H-5′); 8.07–8.11 (m; 2H; H-2′, H-6′). ^13^C NMR (125.7 MHz; DMSO-*d*_6_) *δ* (ppm) 55.5 (C(4′)-OCH_2_); 56.2 (C(7)-OCH_2_); 56.5 (C(5)-OCH_2_); 58.2 (C(3)-OCH_2_); 78.2 (C(7)-OCH_2_C≡CH); 78.5 (C(5)-OCH_2_C≡CH); 78.6 (C(4′)-OCH_2_C≡CH); 78.76 (C(4′)-OCH_2_C≡CH); 78.79 (C(3)-OCH_2_C≡CH); 78.9 (C(5)-OCH_2_C≡CH); 79.05 (C(7)-OCH_2_C≡CH); 79.10 (C(3)-OCH_2_C≡CH); 94.7 (C-8); 98.4 (C-6); 108.8 (C-10); 114.7 (C-3′, C-5′); 123.0 (C-1′); 129.8 (C-2′, C-6′); 137.4 (C-3); 152.7 (C-2); 157.80 (C-9); 157.82 (C-5); 158.8 (C-4′); 161.2 (C-7); 171.9 (C-4). EI-HRMS: M = 438.10893 (delta = −2.0 ppm; C_27_H_18_O_6_).

#### 3.2.8. Click Reaction of 3,7-Bis(*O*-propargyl)kaempferol (**13**) with 4-Fluorobenzyl Azide; Preparation of **15**

**Figure 3:**
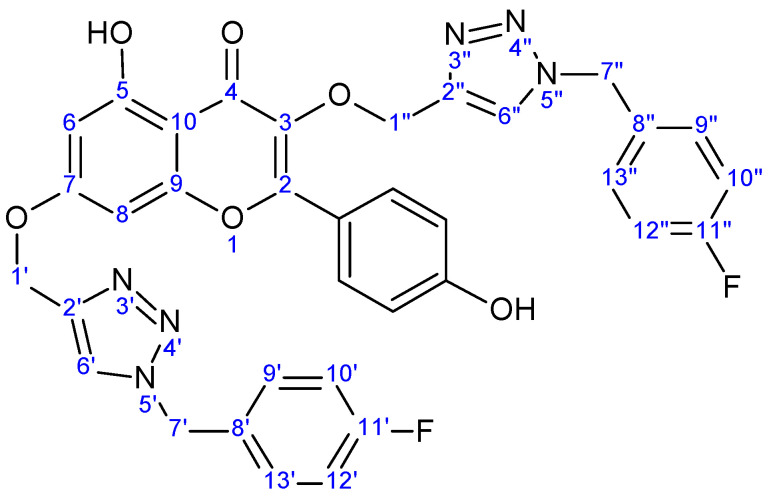
The skeleton numbering of compound **15** used for NMR assignment.

(a) To 3,7-bis(*O*-propargyl) kaempferol (**13**) (140 mg, 0.387 mmol) was added 4-fluorobenzyl azide (117 mg, 0.773 mmol) in toluene solution (10 mL) prepared in situ [30], triphenylphosphine (41 mg, 0.155 mmol), copper(I) iodide (15 mg, 0.077 mmol), 0.43 mL (2.322 mmol) diisopropylethylamine and 13 mL toluene. After reflux for 5.5 hrs, the reaction mixture was diluted with toluene (70 mL), and the mixture was washed with water (45 mL), then the water phase was washed with toluene (15 mL). The combined organic phase was dried with magnesium sulfate and after preparative TLC (dichloromethane-methanol = 20:1) of the residue, 17 mg (7%) product (**15**) was obtained.

(b) To 3,7-bis(*O*-propargyl) kaempferol (**13**) (193 mg, 0.532 mmol) was added 4-fluorobenzyl azide (161 mg, 1.064 mmol) in dichloromethane solution (18 mL) prepared in situ [30], copper(II) sulfate pentahydrate (222 mg, 0.888 mmol), sodium L-ascorbate (351 mg, 1.77 mmol) and water (18 mL). After 8 h of intensive stirring at room temperature, the reaction mixture was diluted with water (70 mL) and extracted with dichloromethane (3 × 80 mL). The combined organic phase was washed with saturated sodium chloride solution (200 mL), and after drying with magnesium sulfate the solution was evaporated. The residue was separated with preparative TLC (dichloromethane-methanol = 20:1) and 18 mg (5%) product (**15**) was obtained (Figure 3). M.p. = 151–153 °C. TLC (dichloromethane-methanol = 20:1); *R_f_* = 0.31. IR (KBr) 1172; 1225; 1512; 1587; 1602; 1665; 3139 cm^−1^. ^1^H NMR (499.9 MHz; DMSO-*d*_6_) *δ* (ppm) 5.20 (s; 2H; H_2_-1′’); 5.27 (s; 2H; H_2_-1′); 5.51 (s; 2H; H_2_-7′’); 5.62 (s; 2H; H_2_-7′); 6.47 (d; *J* = 2.2 Hz; 1H; H-6); 6.85–6.87 (m; 2H; C(2)-Ar: 2x H*_meta_*); 6.88 (d; *J* = 2.2 Hz; 1H; H-8); 7.14–7.19 (m; 2H; H-10′’, H-12′’); 7.19–7.25 (m; 4H; H-10′, H-12′, H-9′’, H-13′’); 7.39–7.44 (m; 2H; H-9′, H-13′); 7.88–7.92 (m; 2H; C(2)-Ar: 2x H*_ortho_*); 8.14 (s; 1H; H-6′’); 8.34 (s; 1H; H-6′); 10.30 (br s; 1H; C(2)-Ar: C*_para_*-OH); 12.68 (s; 1H; C(5)-OH). ^13^C NMR (125.7 MHz; DMSO-*d*_6_) *δ* (ppm) 51.7 (C-7′’); 52.0 (C-7′); 61.7 (C-1′); 64.1 (C-1′’); 93.0 (C-8); 98.3 (C-6); 105.2 (C-10); 115.3 (C(2)-Ar: C*_meta_*); 115.4 (d; ^2^*J*_CF_ = 21.6 Hz; C-10′’, C-12′’); 115.5 (d; ^2^*J*_CF_ = 21.6 Hz; C-10′, C-12′); 120.3 (C(2)-Ar: C_ipso_); 124.9 (C-6′); 125.0 (C-6′’); 129.8 (d; ^3^*J*_CF_ = 8.4 Hz; C-9′’, C-13′’); 130.27 (C(2)-Ar: C*_ortho_*); 130.30 (d; ^3^*J*_CF_ = 8.3 Hz; C-9′, C-13′); 132.1 (d; ^4^*J*_CF_ = 3.0 Hz; C-8′, C-8′’); 135.6 (C-3); 142.1 (C-2′); 142.4 (C-2′’); 156.1 (C-9); 156.5 (C-2); 160.1 (C(2)-Ar: C*_para_*); 160.9 (C-5); 161.4 (d; ^1^*J*_CF_ = 244.2 Hz; C-11′’); 161.7 (d; ^1^*J*_CF_ = 244.4 Hz; C-11′); 163.7 (C-7); 178.0 (C-4). ESI-HRMS: M + H = 665.19497 (delta = −0.7 ppm; C_35_H_27_O_6_N_6_F_2_). HR-ESI-MS-MS (CID = 35%; rel. int. %): 637(51); 620(5); 584(8); 530(20); 512(51); 484(26); 476(100); 456(7); 448(51); 431(8); 299(4).

### 3.3. Biological Evaluation

#### 3.3.1. One-Dose Screen

All compounds were tested initially at a single high dose (10^−5^ *M*) in the full NCI60 cell panel [31,32,33,34,35]. The number reported for the one-dose assay is growth relative to the no-drug control and relative to the time zero number of cells. This allowed the detection of both growth inhibition (values between 0 and 100) and lethality (values less than 0). For example, a value of 100 means no growth inhibition. A value of 30 would mean 70% growth inhibition. A value of 0 means no net growth over the course of the experiment. A value of −30 would mean 30% lethality. A value of −100 means all cells are dead.

#### 3.3.2. Five-Dose Screen

Compounds that exhibited significant growth inhibition in the one-dose screen were evaluated against the 60-cell panel at five concentration levels. The human tumor cell lines of the cancer screening panel were grown in RPMI 1640 medium containing 5% fetal bovine serum and 2 mM l-glutamine. Typically, cells were inoculated in 96-well microtiter plates in 100 μL at plating densities ranging from 5000 to 40,000 cells/well, depending on the doubling time of individual cell lines. After cell inoculation, the microtiter plates were incubated at 37 °C, 5% CO_2_, 95% air, and 100% relative humidity for 24 h prior to the addition of experimental drugs. After 24 h, two plates of each cell line were fixed in situ with trichloroacetic acid (TCA), to represent a measurement of the cell population for each cell line at the time of drug addition (*t*_z_). Experimental drugs were solubilized in dimethyl sulfoxide at 400-fold the desired final maximum test concentration and stored frozen prior to use. At the time of drug addition, an aliquot of frozen concentrate was thawed and diluted to twice the desired final maximum test concentration with complete medium containing 50 μg ml^−1^ gentamicin. Additional four, 10-fold or ½ log serial dilutions were made to provide a total of five drug concentrations plus control. Aliquots of 100 μL of these different drug dilutions were added to the appropriate microtiter wells already containing 100 μL of medium, resulting in the required final drug concentrations.

Following drug addition, the plates were incubated at 37 °C, 5% CO_2_, 95% air, and 100% relative humidity for an additional 48 h. For adherent cells, the assay was terminated by the addition of cold TCA. Cells were fixed in situ by the addition of 50 μL of cold 50% (*w*/*v*) TCA, and incubated at 4 °C for 60 min. The supernatant was discarded, and the plates were washed with water (5×) and dried in air. Sulforhodamine B (SRB) solution (100 μL) at 0.4% (*w*/*v*) in 1% acetic acid was added to each well, and plates were incubated at room temperature for 10 min. After staining, the unbound dye was removed by washing five times with 1% acetic acid, and the plates were dried in the air. The bound stain is subsequently solubilized with 10 mM trizma base, and the absorbance is read on an automated plate reader at λ = 515 nm. Using the seven absorbance measurements [time zero (*t*_z_), control growth (*c*), and test growth in the presence of drug at the five concentration levels (*t*_i_)], the percentage growth was calculated at each of the drug concentration levels. Growth inhibition (%) was calculated as:

[(*t*_i_ − *t*_z_)/(*c* − *t*_z_)] × 100, for concentrations where *t*_i_ ≥ *t*_z_ (1)

[(*t*_i_ − *t*_z_)/(*t*_z_)] × 100, for concentrations where *t*_i_ < *t*_z_. (2)

Three dose-response parameters were calculated as follows. *GI*_50_ (growth inhibition of 50%) was calculated from Equation (3), which is the drug concentration resulting in a 50% reduction in the net protein increase (as measured by SRB staining) in control cells during the drug incubation. The drug concentration resulting in total growth inhibition (TGI) was calculated from Equation (4), where *t*_i_ = *t*_z_. The *LC*_50_ indicating a 50% net loss of cells following treatment was calculated from Equation (5):

[(*t*_i_ − *t*_z_)/(*c* − *t*_z_)] × 100 = 50 (3)

[(*t*_i_ − *t*_z_)/(*c* − *t*_z_)] × 100 = 0 (4)

[(*t*_i_ − *t*_z_)/(*t*_z_)] × 100 = −50. (5)

#### 3.3.3. Antiproliferative Assay on HeLa and SiHa Cells

Cervical adenocarcinoma (HeLa) and cervical carcinoma (SiHa) cells were obtained from the European Collection of Cell Cultures (Salisbury, UK) and the American Type Tissue Culture Collection (Manassas, VA, USA), respectively. The cells were cultured in Minimum Essential Medium supplemented with 10% fetal bovine serum, 1% non-essential amino acids, and 1% penicillin-streptomycin at 37 °C in a humidified atmosphere. Media and supplements were purchased from Lonza Group Ltd. (Basel, Switzerland). Cell viability was assessed by the MTT assay as published before [36]. Briefly, the cells were seeded in 96 well plates at 5000 cells/well density. After 24 h, 100 µL of new media containing the test samples was added. After incubation for 72 h, an aliquot of 44 µL of MTT solution (5 mg/mL) was added. After incubation for a further 4 h, the medium was removed by aspiration, the precipitated formazan crystals were dissolved by adding 100 µL of DMSO to each well, and the plates were shaken at 37 °C for 1 h. The absorbance was measured at 545 nm with a microplate reader. IC_50_ values were calculated by fitting sigmoidal dose–response curves by the nonlinear regression model log (inhibitor) vs. normalized response and variable slope fit of GraphPad Prism 6 (GraphPad Software Inc., San Diego, CA, USA). Clinically utilized anticancer agent cisplatin (Ebewe GmbH, Unterach, Austria) was included as a reference molecule.

## 4. Conclusions

As a result of the current study, hybrid compounds containing chrysin coupled with substituted 1,2,3-triazole pharmacophores showed significant in vitro anticancer activities on several cell lines of different types of cancer. Moreover, the activity of the bis-conjugated derivatives of chrysin was also considerable. Therefore, it may be a reasonable strategy to prepare further hybrid molecules of flavones with more complex structures to obtain potentially valuable new antitumor leads.

## Figures and Tables

**Table 1:** Antiproliferative activities of chrysin (**3**), hybrids **8**–**11** and **15** against 60 human cancer cell lines in vitro. In connection with GPR values, the negative numbers causing cell death are highlighted in bold. Values where *GI*_50_ < 10 µM are highlighted in bold, too.

Type	Growth Percent Rates (GPR) at 10 µM (%), GI_50_ (µM)							
	3	8		9	10		11	15
	GPR	GPR	*GI*_50_	GPR	GPR	*GI*_50_	GPR	GPR
**Leukemia**								
*CCRF-CEM*	102.24	108.83	>100	112.13	77.52	>100	104.65	60.89
*HL-60(TB)*	116.20	103.89	>100	114.55	98.05	>100	111.80	105.09
*K-562*	96.80	109.01	>100	103.64	45.49	>100	97.39	46.23
*MOLT-4*	105.93	95.46	>100	105.64	83.24	>100	96.99	79.61
*RPMI-8226*	101.04	93.67	>100	99.31	45.61	-	116.61	72.72
*SR*	77.87	96.85	-	-	32.75	-	-	64.31
**Non-small cell lung cancer**								
*A549/ATCC*	98.46	81.15	19.6	89.08	23.19	-	96.90	65.31
*EKVX*	89.35	60.13	63.8	90.34	57.19	-	102.98	87.06
*HOP-62*	113.09	**−28.49**	**3.76**	93.12	12.36	**2.33**	91.16	84.75
*HOP-92*	77.93	**−3.35**	**4.43**	94.82	**−17.95**	**1.89**	84.25	44.25
*NCI-H226*	86.84	60.93	**3.51**	75.07	-	**2.07**	-	42.31
*NCI-H23*	92.57	45.78	**7.70**	87.18	29.16	**3.70**	88.96	49.35
*NCI-H322M*	98.30	86.09	>100	93.16	43.54	-	95.03	70.85
*NCI-H460*	98.34	71.35	-	98.72	2.98	-	104.21	49.75
*NCI-H522*	88.95	15.11	**6.60**	87.56	17.59	**3.66**	73.12	48.28
**Colon cancer**								
*COLO 205*	104.94	102.72	>100	102.46	68.37	-	118.35	108.22
*HCC-2998*	102.88	100.11	>100	88.27	74.13	>100	100.57	95.35
*HCT-116*	82.69	48.53	**5.91**	86.67	18.21	**3.52**	88.21	41.04
*HCT-15*	90.99	94.40	-	87.80	73.48	-	96.46	77.80
*HT29*	102.89	100.18	>100	99.75	35.13	-	113.48	100.61
*KM12*	92.93	99.40	>100	100.75	43.34	-	100.81	83.69
*SW-620*	101.60	102.02	>100	99.67	60.30	-	95.28	79.33
**CNS cancer**								
*SF-268*	101.55	14.38	**4.32**	98.12	21.78	**3.52**	89.15	63.29
*SF-295*	99.86	10.37	10.2	96.99	38.91	**2.32**	99.59	65.43
*SF-539*	92.17	1.10	**5.17**	84.22	**−10.54**	**2.21**	88.21	38.91
*SNB-19*	86.04	**−32.68**	**4.51**	86.51	12.08	**4.55**	76.47	54.14
*SNB-75*	88.98	**−65.88**	**3.74**	85.58	6.29	**1.69**	81.23	53.82
*U251*	80.67	**−16.90**	13.9	93.22	10.45	**2.80**	106.25	71.92
**Melanoma**								
*LOX IMVI*	85.08	78.50	>100	95.50	34.84	-	99.64	58.11
*MALME-3M*	101.76	6.44	**5.06**	83.74	15.21	**2.03**	88.16	53.41
*M14*	106.78	65.94	>100	94.81	56.84	-	89.13	55.83
*MDA-MB-435*	99.53	98.22	-	95.76	40.86	-	100.25	67.94
*SK-MEL-2*	109.90	**−39.78**	**6.80**	99.46	48.82	**4.49**	85.75	83.61
*SK-ML-28*	101.70	93.61	-	92.25	29.89	-	100.20	39.71
*SK-MEL-5*	92.85	84.47	>100	93.58	17.09	-	89.29	54.58
*UACC-257*	118.94	86.27	>100	95.41	46.37	-	94.84	83.76
*UACC-62*	82.24	53.15	-	76.28	29.96	-	88.18	42.06
**Ovarian cancer**								
*IGROV1*	95.22	31.22	17.6	78.98	43.47	**4.45**	94.68	53.46
*OVCAR-3*	97.60	61.68	-	112.53	21.16	-	105.28	53.34
*OVCAR-4*	112.07	-	-	97.37	**−9.56**	-	100.27	66.63
*OVCAR-5*	99.07	83.70	>100	95.94	55.10	-	93.98	81.04
*OVCAR-8*	95.19	20.41	**3.76**	91.53	12.68	-	91.22	56.95
*NCI/ADR-RES*	92.84	43.17	**5.57**	78.12	70.43	>100	99.23	88.99
*SK-OV-3*	128.15	20.59	**6.57**	91.10	16.22	-	109.51	87.44
**Renal cancer**								
*786-0*	99.20	2.24	**9.26**	101.61	1.13	**1.96**	100.98	74.81
*A498*	86.62	63.12	42.0	88.60	30.81	-	95.12	71.72
*ACHN*	85.03	5.21	**6.23**	89.78	7.30	-	85.97	53.80
*CAKI-1*	83.56	58.70	**5.76**	96.44	54.63	-	83.45	67.48
*RXF 393*	91.19	9.11	**3.58**	-	**−8.05**	**1.78**	85.59	37.74
*SN12C*	85.77	58.08	>100	98.25	38.67	-	85.22	56.19
*TK-10*	107.21	0.59	10.4	102.72	37.10	**3.01**	106.68	90.47
*UO-31*	89.42	67.00	-	74.94	3.64	-	81.08	79.66
**Prostate cancer**								
*PC-3*	93.17	75.99	-	92.51	35.99	-	107.77	77.08
*DU-145*	92.00	65.83	>100	107.16	40.01	-	101.38	67.54
**Breast cancer**								
*MCF7*	103.05	65.73	-	87.76	53.27	-	88.82	59.60
*MDA-MB-231/ATCC*	82.64	12.02	16.6	82.84	9.10	**2.34**	73.21	57.07
*HS 578T*	92.51	9.08	**6.26**	85.62	20.89	**3.28**	78.08	45.96
*BT-549*	91.01	16.31	**7.98**	83.18	18.75	-	78.58	49.15
*T-47D*	101.44	44.20	-	85.48	40.62	-	96.27	52.68
*MDA-MB-468*	91.09	53.94	20.0	80.62	38.69	**1.97**	74.09	54.70

**Table 2:** In vitro antiproliferative activity of compounds **8** and **10** against human cervical cancer cell lines. Compounds were tested in the concentration range of 0.1–30 µM in 2 biological replicates, 5 parallel measurements each. *IC*_50_ values and their 95% confidence intervals (C.I.) are presented. Value where *IC*_50_ < 1 µM is highlighted in bold. Cisplatin was included as a reference agent.

*IC*_50_ [95% Confidence Interval](µM)		
Hybrid/Cell Line	HeLa	SiHa
8	1.909 [1.543–2.361]	>30
**10**	**0.7331** [0.5771–0.9312]	1.352 [1.148–1.592]
Cisplatin	12.26 [10.36–14.49]	5.305 [4.650–6.053]

## Data Availability

Not available.

## Sample Availability

Samples of the compounds are not available from the authors.

## References

[1] Nepali K., Sharma S., Sharma M., Bedi P.M.S., Dhar K.L. (2014). Rational approaches, design strategies, structure activity relationship and mechanistic insights for anticancer hybrids. Eur. J. Med. Chem..

[2] Choudhary S., Singh P.K., Verma H., Singh H., Silakari O. (2018). Success stories of natural product-based hybrid molecules for multi-factorial diseases. Eur. J. Med. Chem..

[3] Mayer S., Keglevich P., Hazai L. (2022). *Vinca* Hybrids with Antiproliferative Effect. Med. Res. Arch..

[4] Keglevich P., Hazai L., Gorka-Kereskényi Á., Péter L., Gyenese J., Lengyel Z., Kalaus G., Dubrovay Z., Dékány M., Orbán E. (2013). Synthesis and *in vitro* antitumor effect of new vindoline derivatives coupled with amino acid esters. Heterocycles.

[5] Keglevich A., Dányi L., Rieder A., Horváth D., Szigetvári Á., Dékány M., Szántay C., Latif A.D., Hunyadi A., Zupkó I. (2020). Synthesis and Cytotoxic Activity of New Vindoline Derivatives Coupled to Natural and Synthetic Pharmacophores. Molecules.

[6] Keglevich A., Zsiros V., Keglevich P., Szigetvári Á., Dékány M., Szántay C., Mernyák E., Wölfling J., Hazai L. (2019). Synthesis and in vitro Antitumor Effect of New Vindoline-Steroid Hybrids. Curr. Org. Chem..

[7] Mayer S., Nagy N., Keglevich P., Szigetvári Á., Dékány M., Szántay C., Hazai L. (2021). Synthesis of Novel Vindoline-Chrysin Hybrids. Chem. Biodivers..

[8] Keglevich A., Szigetvári Á., Dékány M., Szántay C., Keglevich P., Hazai L. (2019). Synthesis and in vitro Antitumor Effect of New Vindoline Derivatives Coupled with Triphenylphosphine. Curr. Org. Chem..

[9] Mayer S., Keglevich P., Ábrányi-Balogh P., Szigetvári Á., Dékány M., Szántay C., Hazai L. (2020). Synthesis and In Vitro Anticancer Evaluation of Novel Chrysin and 7-Aminochrysin Derivatives. Molecules.

[10] Kopustinskiene D.M., Jakstas V., Savickas A., Bernatoniene J. (2020). Flavonoids as Anticancer Agents. Nutrients.

[11] Ren W., Qiao Z., Wang H., Zhu L., Zhang L. (2003). Flavonoids: Promising Anticancer Agents. Med. Res. Rev..

[12] Xu Z., Zhao S.-J., Liu Y. (2019). 1,2,3-Triazole containing hybrids as potential anticancer agents: Current developments, action mechanisms and structure-activity relationship. Eur. J. Med. Chem..

[13] El Azab I.H., El-Sheshtawy H.S., Bakr R.B., Elkanzi N.A.A. (2021). New 1,2,3-Triazole-Containing Hybrids as Antitumor Candidates: Design, Click Reaction Synthesis, DFT Calculations, and Molecular Docking Study. Molecules.

[14] Liang T., Sun X., Li W., Hou G., Gao F. (2021). 1,2,3-Triazole-Containing Compounds as Anti-Lung Cancer Agents: Current Developments, Mechanisms of Action, and Structure-Activity Relationship. Front. Pharmacol..

[15] Çot A., Çeşme M., Onur S., Aksakal E., Şahin İ., Tümer F. (2022). Rational design of 1,2,3-triazole hybrid structures as novel anticancer agents: Synthesis, biological evaluation and molecular docking studies. J. Biomol. Struct. Dyn..

[16] Pereira D., Pinto M., Correia-da-Silva M., Cidadae H. (2022). Recent Advances in Bioactive Flavonoid Hybrids Linked by 1,2,3-Triazole Ring Obtained by Click Chemistry. Molecules.

[17] Kant R., Kumar D., Agarwal D., Gupta R.D., Tilak R., Awasthi S.K., Agarwal A. (2016). Synthesis of newer 1,2,3-triazole linked chalcone and flavone hybrid compounds and evaluation of their antimicrobial and cytotoxic activities. Eur. J. Med. Chem..

[18] Rao Y.J., Sowjanya T., Thirupathi G., Murthy N.Y.S., Kotapalli S.S. (2018). Synthesis and biological evaluation of novel flavone/triazole/benzimidazole hybrids and flavone/isoxazole-annulated heterocycles as antiproliferative and antimycobacterial agents. Mol. Divers..

[19] Qi Y., Ding Z., Yao Y., Ma D., Ren F., Yang H., Chen A. (2019). Novel triazole analogs of apigenin-7-methyl ether exhibit potent antitumor activity against ovarian carcinoma cells via the induction of mitochondrial-mediated apoptosis. Exp. Ther. Med..

[20] Li X., Cai Y., Yang F., Meng Q. (2017). Synthesis and molecular docking studies of chrysin derivatives as antibacterial agents. Med. Chem. Res..

[21] Mehdi S.H., Nafees S., Zafaryab M., Khan M.A., Rizvi M.M.A. (2018). Chrysin: A Promising Anticancer Agent its Current Trends and Future Perspectives. Eur. J. Exp. Biol..

[22] Khoo B.Y., Chua S.L., Balaram P. (2010). Apoptotic Effects of Chrysin in Human Cancer Cell Lines. Int. J. Mol. Sci..

[23] Mei Q., Wang C., Yuan W., Zhang G. (2015). Selective methylation of kaempferol via benzylation and deacetylation of kaempferol acetates. Beilstein J. Org. Chem..

[24] Agalave S.G., Maujan S.R., Pore V.S. (2011). Click Chemistry: 1,2,3-Triazoles as Pharmacophores. Chem. Asian J..

[25] Bozorov K., Zhao J., Aisa H.A. (2019). 1,2,3-Triazole-containing hybrids as leads in medicinal chemistry: A recent overview. Bioorg. Med. Chem..

[26] Alam M.M. (2022). 1,2,3-Triazole hybrids as anticancer agents: A review. Arch. Pharm..

[27] Tian L., Zheshan Q., Yingquan F., Hongjing Y. (2020). Design, Synthesis and Antiproliferative Activity of Chrysin Derivatives Bearing Triazole Moieties. Chin. J. Org. Chem..

[28] Kolb H.C., Sharpless K.B. (2003). The growing impact of click chemistry on drug discovery. Drug Discov. Today.

[29] Ruiz-Mendoza F.J., Mendoza-Espinoza D., Gonzalez-Montiel S. (2018). Synthesis and Catalytic Activity of Coumarin- and Chrysin-Tethered Triazolylidene Gold(I) Complexes. Eur. J. Inorg. Chem..

[30] Rodriguez-Hernández D., Demuner A.J., Barbosa L.C.A., Heller L., Csuk R. (2016). Novel hederagenin–triazolyl derivatives as potential anti-cancer agents. Eur. J. Med. Chem..

[31] Monks A., Scudiero D., Skehan P., Shoemaker R.H., Paull K., Vistica D., Hose C., Langley J., Cronise P., Vaigro-Wolff A. (1991). Feasibility of a high-flux anticancer drug screen using a diverse panel of cultured human tumor cell lines. J. Natl. Cancer Inst..

[32] Shoemaker R.H. (2006). The NCI60 human tumour cell line anticancer drug screen. Nat. Rev. Cancer.

[33] Alley M.C., Scudiero D.A., Monks A.M., Hursey L., Czerwinski M.J., Fine D.L., Abbott B.J., Mayo J.G., Shoemaker R.H., Boyd M.R. (1988). Feasibility of Drug Screening with Panels of Human Tumor Cell Lines Using a Microculture Tetrazolium Assay. Cancer Res..

[34] Shoemaker R.H., Monks A., Alley M.C., Scudiero D.A., Fine D.L., McLemore T.L., Abbott B.J., Paull K.D., Mayo J.G., Boyd M.R. (1988). Development of Human Tumor Cell Line Panels for Use in Disease-Oriented Drug Screening. Prog. Clin. Biol. Res..

[35] NCI-60 Screening Methodology. https://dtp.cancer.gov/discovery_development/nci-60/methodology.htm.

[36] Latif A.D., Gonda T., Vágvölgyi M., Kúsz N., Kulmány A., Ocsovszki I., Zomborszki Z.P., Zupkó I. (2019). Synthesis and In Vitro Antitumor Activity of Naringenin Oxime and Oxime Ether Derivatives. Int. J. Mol. Sci..

